# Factors Involved in the Collaboration Between the National Comprehensive Cancer Control Programs and Tobacco Control Programs: A Qualitative Study of 6 States, United States, 2012

**DOI:** 10.5888/pcd12.150012

**Published:** 2015-05-28

**Authors:** Behnoosh Momin, Antonio Neri, Sonya A. Goode, Nikie Sarris Esquivel, Carol L. Schmitt, Jennifer Kahende, Lei Zhang, Sherri L. Stewart

**Affiliations:** Author Affiliations: Antonio Neri, Sherri L. Stewart, Division of Cancer Prevention and Control, Centers for Disease Control and Prevention, Atlanta, Georgia; Sonya A. Goode, Nikie Sarris Esquivel, Carol L. Schmitt, RTI International, Research Triangle Park, North Carolina; Jennifer Kahende, Lei Zhang, Office on Smoking and Health, Centers for Disease Control and Prevention, Atlanta, Georgia.

## Abstract

**Introduction:**

Historically, federal funding streams to address cancer and tobacco use have been provided separately to state health departments. This study aims to document the impact of a recent focus on coordinating chronic disease efforts through collaboration between the 2 programs.

**Methods:**

Through a case-study approach using semistructured interviews, we collected information on the organizational context, infrastructure, and interaction between cancer and tobacco control programs in 6 states from March through July 2012. Data were analyzed with NVivo software, using a grounded-theory approach.

**Results:**

We found between-program activities in the state health department and coordinated implementation of interventions in the community. Factors identified as facilitating integrated interventions in the community included collaboration between programs in the strategic planning process, incorporation of one another’s priorities into state strategic plans, co-location, and leadership support for collaboration. Coalitions were used to deliver integrated interventions to the community. Five states perceived high staff turnover as a barrier to collaboration, and all 5 states felt that federal funding requirements were a barrier.

**Conclusions:**

Cancer and tobacco programs are beginning to implement integrated interventions to address chronic disease. Findings can inform the development of future efforts to integrate program activities across chronic disease prevention efforts.

## Introduction

Tobacco control is among the highest priority prevention components to reduce cancer mortality rates and a major modifiable risk factor to control the growing epidemic of chronic diseases (1). In 2014, the US Department of Health and Human Services published a report of the Surgeon General, which highlights the progress of reducing tobacco use and the continuing burden caused by smoking (2). The Centers for Disease Control and Prevention (CDC) established the National Comprehensive Cancer Control Program (CCC) in 1998 and the National Tobacco Control Program (TCP) in 1999 to implement evidence-based cancer and tobacco control interventions.

Collaboration between both programs to leverage existing resources has been encouraged to increase efficiency (3). Although state health departments historically have received federal funding separately for specific chronic disease–oriented programs, CDC’s National Center for Chronic Disease Prevention and Health Promotion (NCCDPHP) is invested in enhancing the relationship between the CCC- and TCP-funded programs. Efforts began in 2005 to integrate chronic disease prevention programs, and in 2008 NCCDPHP released its first collaborative chronic disease funding announcement (4). Although evaluation of the effects of these collaborative efforts has long been a goal, relating program-specific findings to a broader group of CCC- and TCP-funded programs in the United States has been difficult (5).

The concept of collaboration as examined in this study is analogous to integration described in research by Salinsky and Gursky (6), in which integration in chronic disease in public health is defined as the “strategic alignment of chronic disease categorical program resources to increase the effectiveness and efficiency of each program in a partnership without compromising the integrity of categorical program objectives.” Butterfoss and Kegler’s (7) Community Coalition Action Theory (CCAT) is a framework used in public health literature to describe processes that lead to the implementation of integrated strategies by agencies working toward a common outcome, a definition consistent with the description of integration by Salinsky and Gursky. The CCAT model shows that infrastructure components can create “collaborative synergy,” which occurs between 2 or more agencies and is characterized by shared resources, member engagement, and collaborative assessment and planning. High levels of collaborative synergy, in turn, should lead to multi-organizational interventions that are better coordinated, in this case better integration of tobacco control and cancer interventions in the community.

This study used qualitative methods to document key program infrastructure factors, collaborative synergy, and the extent to which 6 programs implemented integrated activities. Factors that hindered or facilitated collaborative synergy and the relationships between collaborative synergy and integrated interventions were also examined.

## Methods

### Theoretical framework and participants

We used a case-study approach to characterize collaboration between funded programs in 6 states. The CCAT model was used as the framework to characterize the organizational attributes, internal organizational activities, and interventions these 2 programs engaged in together.

Participants were staff of the programs in 6 states (Alabama, Arkansas, Delaware, Florida, Nebraska, and Vermont) from March through July 2012. The states were selected using the following criteria: demonstrated ability to conduct activities under both cooperative agreements as defined by CDC, a history of conducting epidemiological research with the ability to designate an epidemiologist who could participate in study activities, innovative tobacco cessation activities in place, and ongoing data collection for the National Quitline Data Warehouse or had a statewide quitline registry. Other states may have met the criteria; however, the states included were those that expressed interest through submission of an application to the study.

To ensure anonymity, states were labeled A through F (Table 1). Participants were selected on the basis of their roles in their program: program directors, health department directors, outreach and media coordinators, epidemiologists, evaluation specialists, and coalition leaders. Each state selected a primary contact person who identified individuals in these roles in their health department. We interviewed 79 participants from all 6 states, individually or in a group (Table 2).

**Table 1:** Characteristics of Participating States, Qualitative Study on State Collaboration for Cancer and Tobacco Control Programs, United States, 2012

State^a^	Programs Are Colocated^b^	Has at Least 1 Staff Person with Experience in Both Programs	CCC and TCP Program Directors Report to Same Supervisor	State Health Department, Local, or Both
A	No (but previously were)	No	No	State + local
B	Yes	Yes	Yes	State + local
C	No	No	No	Only state
D	No	Yes	No	State + local
E	No	No	No	Only state
F	Yes	Yes	Yes	Only state

Abbreviation: CCC, National Comprehensive Cancer Control Program; CDC, Centers for Disease Control and Prevention; TCP, National Tobacco Control Program.

a All states were 2010 CDC Coordinating Chronic Disease Grant Recipients. For anonymity purposes, state names are not displayed.

b Because the terms with which each state health department describe their organizational structure vary (ie, a “bureau” in one organization could be at the same level as a “center” at another organization), we indicate whether CCC and TCP programs are co-located. We defined “co-located” as within the same department at the lowest level.

**Table 2:** Number of Participants Interviewed at Each Site and Organization Represented, Qualitative Study on State Collaboration for Cancer and Tobacco Control Programs, United States, 2012

Site	HD	CCC	TCP/QL	Coalition	Other^a^	Total
State A	2	6	9	5 (3 CCC, 2 TCP)	0	22
State B	3	4	4	2 (2 CCC)	0	13
State C	4	4	6	4 (2 CCC, 2 TCP)	1	19
State D	1	2	4	1 (1 CCC)	0	8
State E	1	3	4	2 (1 CCC, 1 TCP)	1	11
State F	2	2	5	2 (1 CCC, 1 TCP)	1	12
Total	13	21	32	16	3	85

Abbreviations: CCC, National Comprehensive Cancer Control Program; HD, health department; QL, Quitline; TCP, National Tobacco Control Program.

a Other interviewees were an epidemiologist, a media coordinator, and a former coalition chair.

### Data collection

We used the CCAT model to develop the interview guides. As a result, the guides included questions relating to organizational characteristics, evidence of collaborative synergy between organizations, and the extent to which cancer and tobacco control programs integrated interventions. Interviewees were also asked to describe perceived barriers and facilitators to collaboration. Although the interview guides were developed and tailored to the type of interviewee (leadership, CCC and TCP staff), examples of similar questions asked of all interviewees include the following: 1) Do the CCC or TCP programs sit in the same division of the health department? 2) Are there ways in which the health department leadership requires programs to work together? and 3) Are there any barriers that affect TCP’s ability to communicate with the CCC staff?

A designated contact person recruited and scheduled all interviewees, and no incentives were offered. A combination of individual in-person and telephone interviews was conducted, as were 16 in-person group interviews. The 6 telephone interviews were scheduled with staff members who were unavailable to meet in person. The interview team consisted of an interviewer and a note-taker. Each interview was recorded. The lengths of the individual interviews (n = 43) varied from 30 minutes to 1 hour. Sixteen group interviews (n = 42) lasted approximately 1.5 hours and included 2 to 5 participants per group. Group interview participants were selected on the basis of scheduling availability and the extent to which they shared roles in a program. One group interview consisted of both leadership and program staff members.

### Data analysis

Notes and interview transcriptions were analyzed using NVivo software (8). Other secondary data sources, including state organizational charts and state cancer plans, were also imported into NVivo. For analysis we used a grounded-theory approach (9). A list of codes was developed and reviewed by 2 independent coders. One coder attended all site visits, and the other did not attend any. Both coders met regularly to review the codes and ensure that they had a similar understanding of the context. If there was a discrepancy, a third coder reviewed the data, determined the most appropriate code, and developed a decision rule with the other 2 coders. For 20% of the interviews, a third person double-coded the data. We obtained an average interrater agreement of 90% or above across all double-coded data.

## Results

Participants described 2 categories of activities that were consistent with collaborative synergy (conducted within the organization) and integrated program interventions in the community (Table 3). Participants mentioned a greater number of collaborative activities consistent with the synergy construct than those consistent with integrated interventions.

**Table 3:** Key Findings Related to Collaborative Activities Conducted in the Organization and at the Point of Service, Qualitative Study on State Collaboration for Cancer and Tobacco Control Programs, United States, 2012^a^

Site	Collaborative Activities Conducted in the Organization			Collaborative Activities Conducted at Point of Service			
	Informal Communication	Formal Communication	Strategic Planning	Networked Partnerships	Policy and Systems Change Strategies	Health Communications	Cross-Promotion
A	X	X	X	X	X	X	X
B		X	X	X		X	
C	X	X	X	X		X	
D		X	X		X		
E	X	X	X	X	X		X
F	X	X	X	X	X		X

a X denotes that a state is engaged in the specified activity. Federal funding was received through the National Comprehensive Cancer Control Program and the National Tobacco Control Program.

### Collaborative synergy within the health department

Between-program activities consistent with collaborative synergy included staff engagement (informal and formal communications) and assessment and planning (collaborative strategic planning). Informal communication describes the casual conversations that occurred naturally in the workplace between the 2 programs. Program staff described formal communication as email distributions, including updates on quitline activities to both CCC and TCP staff, use of a listserv, or regular joint meetings. All 6 states described both formal and informal communication between the programs.

Strategic planning involved developing formal opportunities for future integration of CCC and TCP activities in the community. One example of strategic planning is codevelopment, which is defined as the 2 programs contributing to each other’s state plans and joint grant writing. One state participant acknowledged that the state plan was not developed in as collaborative a manner as the participant had hoped; therefore, the participant intended to increase collaborative development of plans in the future. Two state participants described a long history of joint grant writing between CCC and TCP staff. However, one state participant viewed joint funding opportunities as an artificial means for future collaborations. The state was referring to a recent funding opportunity (10) that included CCC and TCP and aimed to improve coordination across chronic disease programs; however, this state said that its programs still function on their own as opposed to being integrated.

### Integrated interventions in the community

Participants described 4 types of interventions they conducted collaboratively in the community: networked partnerships, policy and systems change, health communication, and cross-promotion of programs.

All networked partnerships in this study were coalitions. Coalitions typically include public, private, governmental and nongovernmental partners, youth-focused tobacco prevention organizations, local hospitals, and health care organizations. Participants reported that coalitions expand the reach of their programs; it appeared that the coalition was the main structure through which integrated interventions were implemented. In almost every state (n = 5), staff from one program served on the other program’s coalition. One state’s TCP conducted several presentations about the importance of protection from secondhand smoke at the cancer coalition’s state conference. Another state encouraged streamlining coalitions into one overarching council that could coordinate efforts across programs.

Strategies to influence policy refer to the joint activities CCCs or TCPs implement, such as community awareness campaigns about policies that will be voted on or implemented or providing data to inform the public health benefit of policy interventions. One state’s TCP developed a fact sheet on the economic and public health impacts of a cigarette tax. The fact sheet was used by the state’s cancer coalition to inform key decision makers about the impact of a proposed tax increase. Systems change was another activity identified by 4 states. One state described plans to work with a subcontractor to update electronic health records to collect a more comprehensive list of chronic disease indicators

Sharing health communication and marketing materials was perceived as an easy way to integrate services in the community. For example, one TCP-funded program developed promotional postcards and brochures with both cancer and tobacco-use prevention information. One state’s CCC mentioned that a chronic disease multiprogram brochure — including cancer and tobacco control — is being developed. Two states described cross-promotion of activities as being centered on the state quitline, where the CCC website included the quitline telephone number and a link to the TCP website.

### Barriers and facilitators to collaboration

Characteristics of the program, including funding sources, organizational processes, staffing, and leadership, were perceived as barriers or facilitators to CCC and TCP collaborative synergy and integration of activities at the point of service.

Managed resources refer to the funding streams and allocation of money and staff across programs, which are affected by state funding requirements, federal funding requirements, trust regarding financial motives, and mutual funding benefit. State funding requirements were perceived as a barrier to collaboration between the programs in all states. Staff from all 6 states agreed that federal funding requirements hindered both collaborative synergy and integration of interventions. Trust regarding financial motives was also seen as a barrier; in one state, the TCP viewed another program’s collaboration request as possibly motivated by a desire to access the TCP’s funds. Mutual funding in this study refers to the ability of one program’s funding to serve another program’s benefit. One state received funding for a project that allowed for cross-program collaboration with the pooling of resources and viewed this as a facilitator. The state plans were a facilitator to conducting integrated interventions. For example, in some states the CCC-funded program included tobacco control strategies in its cancer plan and was working toward coordinating both programs’ work plans.

Guidelines and recommendations found in the cancer plans consist of the integration of tobacco and cancer control state plans and the creation of state cancer plan subcommittees. They refer to the cancer and tobacco control goals and objectives of each program and the federal and state goals, objectives, and funding priorities that may affect the work of each program. The integration of tobacco and cancer control into each other’s state plans is not necessarily a predictor of integrated activities at the point of service. This was the case in one state where, despite having an integrated state health plan, the 2 programs did not work together. However, some states did collaborate in planning and perceived it as a facilitator of integration. Another perceived facilitator of integration was the creation of subcommittees that included members of both programs with the goal of moving toward addressing chronic disease risk factors.

Infrastructure could be a facilitator or barrier to collaborative synergy. It refers to staffing, the extent to which programs were co-located (within the organizational chart and physical proximity), and the characteristics of program and health department leadership. Physical proximity (ie, offices in the same building or on the same floor) was presumed to be a facilitator of informal communications. Leadership and staffing was a main component under infrastructure, as it refers to the vision of the state health department. A lack of unified or overall leadership support was identified as a barrier. Staff in one state reported that there were 2 leadership perspectives about integration of CCC and TCP efforts. One state reported that the different leadership styles of TCP and CCC administrators led the programs in 2 directions, making it difficult to share resources and ultimately activities. For example, the state TCP was focused on policy strategies and cessation; however, the state CCC was focused on direct services. In another state, although both programs’ staffs were interested in collaborating, upper management did not appear to support collaboration; an opportunity for co-location of both programs in the same bureau was rejected.

Conversely, leadership support was seen as a way to facilitate collaborative synergy between both programs. In multiple states, staff agreed that the leadership must define collaboration. Similarly, in states where program leaders had worked in each other’s programs, leadership support for collaboration was more readily noticed among program staff.

High staff turnover was often noted as a barrier, leading to vacancies and a loss of institutional memory. Two-thirds of the states had problems with staff turnover or hiring freezes. One respondent explained that turnover made it difficult to know who the state’s partners were in the other program, thereby decreasing staff engagement in co-program efforts. However, several interviewees viewed high turnover as a facilitator to collaboration, because it pushed programs to combine resources to achieve their goals.

Overall, the location of programs in separate office spaces and bureaus and divisions seemed to act as a barrier to collaboration, which is consistent with the notion that collaborative synergy between programs is facilitated by engaged staff members. Separate spaces made it difficult to see other program staff regularly and limited the opportunities for formally or informally reviewing each other’s materials or reports.

## Discussion

We found that CCC- and TCP-funded state programs are building collaborative synergy and beginning to integrate their program interventions. This effort is a shift from the traditional “siloed” approach of program implementation that was associated with categorically funded chronic disease prevention programs. We also found that programs were engaging in more collaborative activities consistent with collaborative synergy than those indicative of interventions integrating both program priorities. 

Other findings consistent with the constructs and relationships in the CCAT model (7) highlight actions that the federal government and state health departments could take to create an organizational climate more supportive of program collaborative synergy and integrated interventions. For example, leaders who are educated about the importance of chronic disease interventions and who are trained to integrate them could create the climate that staff from 5 of the 6 states described. In these states, leadership support was perceived as a facilitator of interaction between programs, a finding consistent with both the CCAT model and a large literature focused on public health infrastructure (11). High staff turnover is generally considered to have negative effects in the workplace (12) and was perceived as a barrier to collaboration from staff in 5 states because of its negative effects on interpersonal relationships (13,14) and on the consistency of program direction when it occurred at the leadership level. Likewise, organizational structure was a facilitator of staff engagement when programs were co-located and could be a barrier when they were not. Financial resources are barriers to collaborative synergy when reporting requirements minimize opportunities for staff engagement and co-planning activities. Financial resources can also be a direct barrier to integrated interventions when specific activities are prescribed. Three states perceived collaboration in strategic planning — particularly the integration of tobacco and cancer control priorities into each other’s plan — as a facilitator to integrated activities. Clearly, leadership that supports collaborative programs, along with an organizational structure that limits staff turnover and provides opportunities for staff communication, could create a climate supportive of the type of collaborative synergy that leads to increased integration of chronic disease program interventions.

Of particular interest in our findings was the use of partnerships as the most frequently cited mechanism through which integrated interventions are implemented. It would be of interest to explore whether coalitions focused more broadly on chronic disease prevention could better promote integration of chronic disease interventions while ensuring that the interests of individual program staff are maintained.

Collaborative processes can strengthen existing networks and partnerships and also may encourage the development of new links among organizations from various sectors and levels (15–17). Integrated chronic disease prevention is an approach that targets more than one risk factor or disease outcome, more than one level of influence, more than one disciplinary perspective, more than one type of research method, or more than one societal sector, and it targets populations rather than individuals (15). Despite the barriers to collaborations, staffs from both programs were willing to work together.

This study has 3 limitations. First, the small sample size may limit generalizability. Although the participants reflected a diverse range of roles in the programs, they may not be representative of those in other states. Second, data are subjective and could not always be verified against secondary sources. Finally, during the one group interview at which program leadership and staff were both present, this power dynamic may have produced undue bias in participant responses.

Cancer and tobacco programs are beginning to implement integrated interventions to address chronic disease. Findings can inform the development of future efforts to integrate program activities across chronic disease prevention efforts, and if staff engagement and co-development of strategic plans continue, the quantity of integrated interventions should increase.
